# Advances in Wood Composites II

**DOI:** 10.3390/polym12071552

**Published:** 2020-07-13

**Authors:** Antonios N. Papadopoulos

**Affiliations:** Laboratory of Wood Chemistry and Technology, Department of Forestry and Natural Environment, International Hellenic University, GR-661 00 Drama, Greece; antpap@for.ihu.gr

The main advantage of wood composites is that they can be designed for specific performance requirements or specific qualities, since they are man-made. Therefore, they can be used in a very wide array of applications, from small-home to industrial-scale projects. This, in turn, enables many more options in design without sacrificing structural requirements. At the same time, their ability to be tailored to a number of uses makes them a very viable option for reducing the use of solid wood. This Special Issue, Advances in Wood Composites II, is a continuation of the “Advances in Wood Composites I” [1] and presents recent progress in the enhancement and refinement of the performance and properties of wood composites. This Special Issue, with a collection of 13 original contributions, provides selected examples of recent Advances in Wood Composites.

An excellent and updated review on wood composites and the polymer binders used for their manufacture is published in this Special Issue [2]. The review focuses on the most important aspects to look out for in manufacturing excellent wood composites and on binders that currently dominate in industry. The enormous progress made in this field during the last few years is highlighted, and an exciting and fascinating research future is expected.

It is known that the two major drawbacks of wood composites are their dimensional instability and their susceptibility to biotic and abiotic factors [3,4]. These can be as addressed by the chemical or thermal modification of the raw material [5,6,7,8,9,10,11,12,13,14,15,16,17]. An alternative means, probably more attractive, is so-called nanotechnology. Nanomaterials have unique characteristics, penetrate effectively and deeply into the wood substrate and improve fundamental properties [18,19,20,21,22,23,24,25,26,27,28,29,30,31].

Wollastonite, a silicate mineral, was applied in order to fortify urea formaldehyde resin for the manufacture of two types of composites (medium density fiber board and particleboard) made from chicken feather and wood [32]. It was reported that wollastonite behaved as a reinforcing filler, and therefore, most of the physicomechanical properties of the boards were improved.

In another study, nano-wollastonite and graphene were mixed in a water-based paint in order to investigate the fire properties of beech wood [33]. The results indicated that graphene presents a high potential to be applied as a fire retardant in the protection of wood and wood composites.

An interesting study investigated the performance of wood treated with ammonium hydroxide [34]. It is known that the dark color of wood in ammonia fuming is due to reactions among wood compounds and ammonia gas. These chemical reactions affect the physicomechanical properties of the treated wood. The study demonstrated that ammonia can be successfully used to change the uneven color of black locust wood.

Xia et al. [35] used feather as a source of protein in combination with copper and boron salts, and they made wood preservatives using nano-hydroxyapatite or nano-graphene oxide as nano-carriers. The results revealed that the penetration into the wood structure was successful, and it was concluded, based on the decay experiments, that the protein-based preservatives had the potential to be considered as a low-cost and environmental friendly alternative for wood preservation.

Bekhta et al. [36] studied the effects of selected process variables on temperature evolution and bond quality during the hot pressing of plywood. Their goal was to present an optimization of the manufacturing process in order to reduce both energy and adhesive consumption. The main finding of the study was that the use of densified veneers can reduce the press cycle (time and pressure) and resin consumption. In the same context, they also studied the possible effects of the veneer drying temperature on formaldehyde emission and on key board properties [37]. The results indicated that when elevated temperatures are applied in the drying of veneers, the formaldehyde emission from the boards is reduced significantly. It was concluded that a 185 °C in-steam dryer could be considered as optimum for industrial applications.

The sound absorption properties of wood composites have received limited attention by researchers to date. Noise is a fundamental issue today, especially in big cities. The paper by Tudor et al. [38] tackled this issue. They manufactured insulation panels from bark and studied their sound absorption properties. Bark from spruce and larch was used. They concluded that bark can successfully be used for the manufacture of insulation panels and may substitute materials currently used in sound applications.

Wood plastic composites receive great attention in this Special Issue. This interesting field is a combination of processing techniques for filler and fiber preparation and polymer science. In this context, the application of wood plastic composites in marine applications sounds very interesting. Alrubaie et al. [39] presented an innovative wood plastic composite lumber made from thermally modified wood. Its overall performance was compared to the performance of high-density polyethylene lumber (HDPE). The main conclusion of this study was that this innovative type of composite could successfully replace HDPE in structural applications.

In the area of composites, Cracium et al. [40] presented a natural-fiber-reinforced eco-composite. This was based on wood wastes (obtained by electron beam irradiation) and on a rubber monomer (ethylene-propylene-diene monomer). Sawdust was incorporated as a filler in order to improve the mechanical and physical characteristics of the composite. It was reported that 300 kGy irradiation seemed to be sufficient for obtaining the effect of reinforcement in the composite.

Kumar et al. [41] prepared injection-molded biocomposites in a biopolymeric matrix using raw material from short-rotation species, such as aspen and willow. This study highlighted the technical feasibility of preparing such a type of biocomposite and concluded that short-rotation species have the potential to be an alternative for biocomposite manufacture.

Another interesting topic addressed in this issue is the transparency of wood. Wu et al. [42] used the orthogonal test method to find the best way of partly delignifying wood. They concluded that this type of transparent wood retained most of the wood texture and color and had a certain degree of light, which means that it may used as a functional decorative material.

Last but not least, the issue of waterlogged archeological wood is addressed. Such studies are limited in the literature. Han et al. [43] addressed this issue, in a case study in China, by studying the hygroscopic properties of less decayed and moderately decayed waterlogged archeological wood, collected from marine shipwrecks. It was found that moderately decayed wood possessed higher hygroscopicity than that of less decayed wood. Based on the results of this study, which measures are necessary for shipwreck restoration can be decided.

It can surely and definitely be said that the field of wood composites is a fascinating one and has a bright future. What is presented here and in the previous editorial of the first series of “Advances in Wood Composites” is only a very short overview of what will happen in the near future [1]. Progress and recent developments in this field have been accelerating, and new approaches and ideas are continuously increasing, implying an exciting and interesting research future. Therefore, after the successful Special Issues “Advances in Wood Composites I” and “Advances in Wood Composites II”, which both collected innovative papers from well-known scientists worldwide, a third part of this series is now available and open for submission.
